# Active afforestation of drained peatlands is not a viable option under the EU Nature Restoration Law

**DOI:** 10.1007/s13280-024-02016-5

**Published:** 2024-05-02

**Authors:** Gerald Jurasinski, Alexandra Barthelmes, Kenneth A. Byrne, Bogdan H. Chojnicki, Jesper Riis Christiansen, Kris Decleer, Christian Fritz, Anke Beate Günther, Vytas Huth, Hans Joosten, Radosław Juszczak, Sari Juutinen, Åsa Kasimir, Leif Klemedtsson, Franziska Koebsch, Wiktor Kotowski, Ain Kull, Mariusz Lamentowicz, Amelie Lindgren, Richard Lindsay, Rita Linkevičienė, Annalea Lohila, Ülo Mander, Michael Manton, Kari Minkkinen, Jan Peters, Florence Renou-Wilson, Jūratė Sendžikaitė, Rasa Šimanauskienė, Julius Taminskas, Franziska Tanneberger, Cosima Tegetmeyer, Rudy van Diggelen, Harri Vasander, David Wilson, Nerijus Zableckis, Dominik H. Zak, John Couwenberg

**Affiliations:** 1 Institute of Botany and Landscape Ecology, University of Greifswald, Partner in the Greifswald Mire Centre, Greifswald, Germany; 2 Department of Biological Sciences, Faculty of Science and Engineering, University of Limerick, Limerick, Ireland; 3 Laboratory of Bioclimatology, Poznan University of Life Sciences, Poznan, Poland; 4 Department of Geoscience and Natural Resource Management, University of Copenhagen, Copenhagen, Denmark; 5 Research Institute for Nature and Forest, Brussels, Belgium; 6 Department of Aquatic Biology and Environmental Sciences, RIBES, Radboud University Nijmegen, Nijmegen, The Netherlands; 7 Faculty of Agricultural and Environmental Sciences, University of Rostock, Rostock, Germany; 8 Faculty of Biological and Environmental Sciences, University of Helsinki, Helsinki, Finland; 9 Department of Earth Sciences, University of Gothenburg, Gothenburg, Sweden; 10 Faculty of Forest Sciences and Forest Ecology, University of Göttingen, Göttingen, Germany; 11 Department of Plant Ecology and Environmental Conservation, University If Warsaw, Warsaw, Poland; 12 Institute of Ecology and Earth Sciences, University of Tartu, Tartu, Estonia; 13 Climate Change Ecology Research Unit, Adam Mickiewicz University, Poznań, Poland; 14 Sustainability Research Institute, University of East London, London, UK; 15 Laboratory of Climate and Water Research, Nature Research Centre, Vilnius, Lithuania; 16 Institute for Atmospheric and Earth System Research, University of Helsinki, Helsinki, Finland; 17 Bioeconomy Research Institute, Vytautas Magnus University, Akademija, Lithuania; 18 Department of Forest Sciences, University of Helsinki, Helsinki, Finland; 19 Michael Succow Foundation, Partner in the Greifswald Mire Centre, Greifswald, Germany; 20 School of Biology and Environmental Science, University College Dublin, Dublin, Ireland; 21 Laboratory of Flora and Geobotany, Institute of Botany, Nature Research Centre, Vilnius, Lithuania; 22 Foundation for Peatland Restoration and Conservation, Vilnius, Lithuania; 23 Institute of Geosciences, Faculty of Chemistry and Geosciences, Vilnius University, Vilnius, Lithuania; 24 Geobiology Research Group, Department of Biology, University of Antwerp, Antwerpen, Belgium; 25 Earthy Matters, Donegal, Ireland; 26 Institute of Ecoscience, Aarhus University, Aarhus, Denmark; 27 Leibniz-Institute of Freshwater Ecology and Inland Fisheries Berlin, Berlin, Germany

**Keywords:** Carbon storage, GHG emissions, Nature based solutions, Nature restoration law, Peatland forestry, Peatland restoration

## Abstract

The EU Nature Restoration Law (NRL) is critical for the restoration of degraded ecosystems and active afforestation of degraded peatlands has been suggested as a restoration measure under the NRL. Here, we discuss the current state of scientific evidence on the climate mitigation effects of peatlands under forestry. Afforestation of drained peatlands without restoring their hydrology does not fully restore ecosystem functions. Evidence on long-term climate benefits is lacking and it is unclear whether CO_2_ sequestration of forest on drained peatland can offset the carbon loss from the peat over the long-term. While afforestation may offer short-term gains in certain cases, it compromises the sustainability of peatland carbon storage. Thus, active afforestation of drained peatlands is not a viable option for climate mitigation under the EU Nature Restoration Law and might even impede future rewetting/restoration efforts. Instead, restoring hydrological conditions through rewetting is crucial for effective peatland restoration.

## Introduction

The EU Nature Restoration Law is critical for the restoration of degraded ecosystems and the provision of climate protection, as clearly shown by the most recent IPCC report (IPCC AR6 SYR 2023). The carbon dioxide (CO_2_) sinks necessary to reach climate neutrality and then net cooling during the second half of this century heavily rely on the land use sector (LULUCF), because improved land stewardship is the most mature CO_2_ removal method (e.g. ibid., Field and Mach 2017). However, sinks should not be used to compensate for avoidable sources and emission reduction remains the utmost priority because the sinks cannot—by far—compensate for the large anthropogenic emissions, which urgently need “aggressive and rapid greenhouse gas emission reduction in all sectors of the economy” (Seddon et al. 2021).

Several EU member states pursue the recognition of active afforestation of drained and degraded peatlands (without rewetting) as a measure under the Nature Restoration Law (NRL). This is, however, problematic from a scientific perspective. Firstly, afforestation of drained peatlands, while keeping them drained, will not restore the peatland ecosystem with its flora, fauna and functions. Secondly, as long as a peatland remains drained, it will degrade further, contribute to climate warming through high emissions of CO_2_ and nitrous oxide, and downstream systems may be severely polluted with nutrients and dissolved organic matter (Zak and McInnes 2022). Typically, in forested northern peatlands, much more carbon is stored in the peat (22.6–66.0 kg m^−2^) than in the forest biomass (2.8–5.7 kg m^−2^) (Beaulne et al. 2021). Long-term carbon losses from the drained peatland will likely be larger than the amount of carbon stored in the forest biomass (Dunn and Freeman 2011; Makrickas et al 2023). Carbon will be released to the atmosphere as CO_2_ when the trees or the manufactured timber products reach the end of their life cycle. Actions focussed on a single ecosystem service too often result in adverse outcomes for the ecosystem as a whole.

Presently, the claim that afforestation on drained peatlands could be beneficial for climate change mitigation in the long-term has to be questioned. In most cases, CO_2_ release from peat soil degradation will likely exceed carbon sequestration in the forest biomass when full growth cycles are considered, as was also concluded by the IPCC in the 2013 Wetlands Supplement (IPCC 2014). In contrast, ecosystem-scale flux measurements show that rewetting/restoration^1^ of forestry-drained peatlands can reduce soil CO_2_ emissions and even restore the CO_2_ sink function within a few years/decades (Hambley et al. 2019). A comprehensive assessment must cover the entire land use cycle—from site preparation to sowing or planting, growth of the biomass, thinning or ditch cleaning, through to harvest, the fate of the biomass (whether used for long-lived products or not) and the fallow time before renewed site preparation. For cropping agriculture, this cycle commonly takes in a single year, while in forestry it takes much longer. Longer, comprehensive greenhouse gas monitoring studies are currently only available from the boreal region and even here, the authors call for longer-term studies that include the entire life cycle (*e.g.*, Bjarnadottir et al. 2021). Such studies do not exist to date and so we can only approach the true climate balance of peatland forestry by using space-for-time substitution and stitching together studies that cover different stages of the forestry life cycle.

Here, we review the relevant literature and discuss the role of peatlands in relation to the climate with respect to forestry-drained peatlands in particular. We identify knowledge gaps and suggest future research that will enable better knowledge of the climate effect of peatland forestry.

## Peatlands and climate

A peatland is an area with a naturally accumulated layer of peat at the surface. Peat is a sedentary material consisting of at least 30% (dry mass) of dead organic material. Plants absorb CO_2_ and store carbon in their biomass. When they die, they are decomposed and the CO_2_ is released again. In mires—peatlands that actively accumulate peat^2^—water saturation of the soil effectively excludes oxygen, thereby inhibiting the full decomposition of the plant litter, which then accumulates as peat. In this way, peatlands have sequestered huge amounts of carbon over millennia. Globally, peatlands store approximately 600 Gt of carbon (Yu et al. 2010; UNEP 2022), which is more than is contained in global forest above-ground biomass (Santoro et al. 2021), and have cooled the planet by approximately 0.6 °C over the past 10,000 years (Frolking and Roulet 2007; Joosten et al. 2016). Forests and peatlands are fundamentally different in terms of carbon cycling over time, in that pristine mature forests are generally in balance (although this view has also been challenged by, e.g. Luyssaert et al. (2008)); whereas, peatlands continue to accumulate carbon, year after year.

While mires sequester carbon from the atmosphere, incomplete decomposition under water-saturated oxygen-free conditions results in the production of methane (CH_4_). The amount of CH_4_ released from pristine, wet peatlands varies strongly depending on environmental conditions such as pH, temperature and vegetation (e.g. Lai 2009). Methane is a short-lived greenhouse gas (GHG) and stays in the atmosphere, on average, for less than 12 years. Thus, given steady emissions of CH_4_, a dynamic equilibrium will establish over time in which in a certain year the same amount of CH_4_ will disappear from the atmosphere as is added from the peatland, and the CH_4_ concentration in the atmosphere and the climate impact do not increase any further (Frolking and Roulet 2007). Pristine, undrained peatlands almost always release CH_4_, but the net uptake of CO_2_ overcompensates for the CH_4_ losses in the long-term. In mires, i.e. wet peat-forming peatlands, formation and emissions of nitrous oxide (N_2_O, a potent greenhouse gas) are negligible.

When peatlands are drained, the upper soil layers are no longer water-saturated, oxygen enters the peat, and decomposition of organic matter becomes much more efficient, leading to mineralisation of the peat and, thus, high CO_2_ (Ojanen et al. 2010; Jovani-Sancho et al. 2018) and N_2_O emissions (Klemedtsson et al. 2005; IPCC 2014; Leppelt et al. 2014; Minkkinen et al. 2020). In contrast, CH_4_ production and emissions generally decrease because of water table drawdown; while, drainage ditches may remain a major source of CH_4_ emissions (Minkkinen et al. 1997; Köhn et al. 2021; Rissanen et al. 2023). Draining reduces soil CH_4_ emissions but increases soil CO_2_ and N_2_O emissions, as well as CH_4_ emissions from drainage infrastructure such as ditches. In the case of forestry, drainage also increases tree stand carbon stock, until the stand is cut. This may lead to short-term cooling in the boreal climate zone, but in most cases, the long-term effect will be warming (Laine et al. 1996; Ojanen and Minkkinen 2020).

In order to determine the climate effect of rewetted peatlands, we need to compare CO_2_, CH_4_ and N_2_O emissions, and lateral losses of carbon and nitrogen both in drained and rewetted situations. Rewetting is always a choice between continued emissions of long-lived GHGs (CO_2_ and N_2_O) versus emissions of a short-lived GHG (CH_4_). In the short-term, rewetting will not result in net cooling because of increased CH_4_ emissions, but will in the long-term (decades to centuries) (Wilson et al. 2016, 2022; Günther et al. 2020; Ojanen and Minkkinen 2020). However in most cases, rewetting peatlands will pay off immediately, or after a very short time period, *i.e.*, total radiative forcing is lower than in the drained state (Günther et al. 2020; Tanneberger et al. 2021b). Re-introduction of vegetation on cutover bogs (Rochefort et al. 2003; González and Rochefort 2014), but also after topsoil removal on formerly drained grassland on bog peat (Rosinski et al. 2021) can speed-up restoration of the GHG sink function after rewetting (Nugent et al. 2018, 2019; Huth et al. 2022).

## Forestry on peatlands

Peatland forestry is common in the boreal zone, but also occurs across the temperate climate zone. Globally, approximately 12 million hectares (Mha) of peatlands have been drained for forestry (Minkkinen et al. 2023). This represents approximately 3% of the global peatland area (ca. 440 Mha, Yu et al. 2010). Most of the area under peatland forest is situated in Fennoscandia and Russia, where over 10 Mha of peatlands have been drained for forestry (ibid.). In Canada and the United States, forestry is also practised on undrained peatlands.

**Fig. 1:**
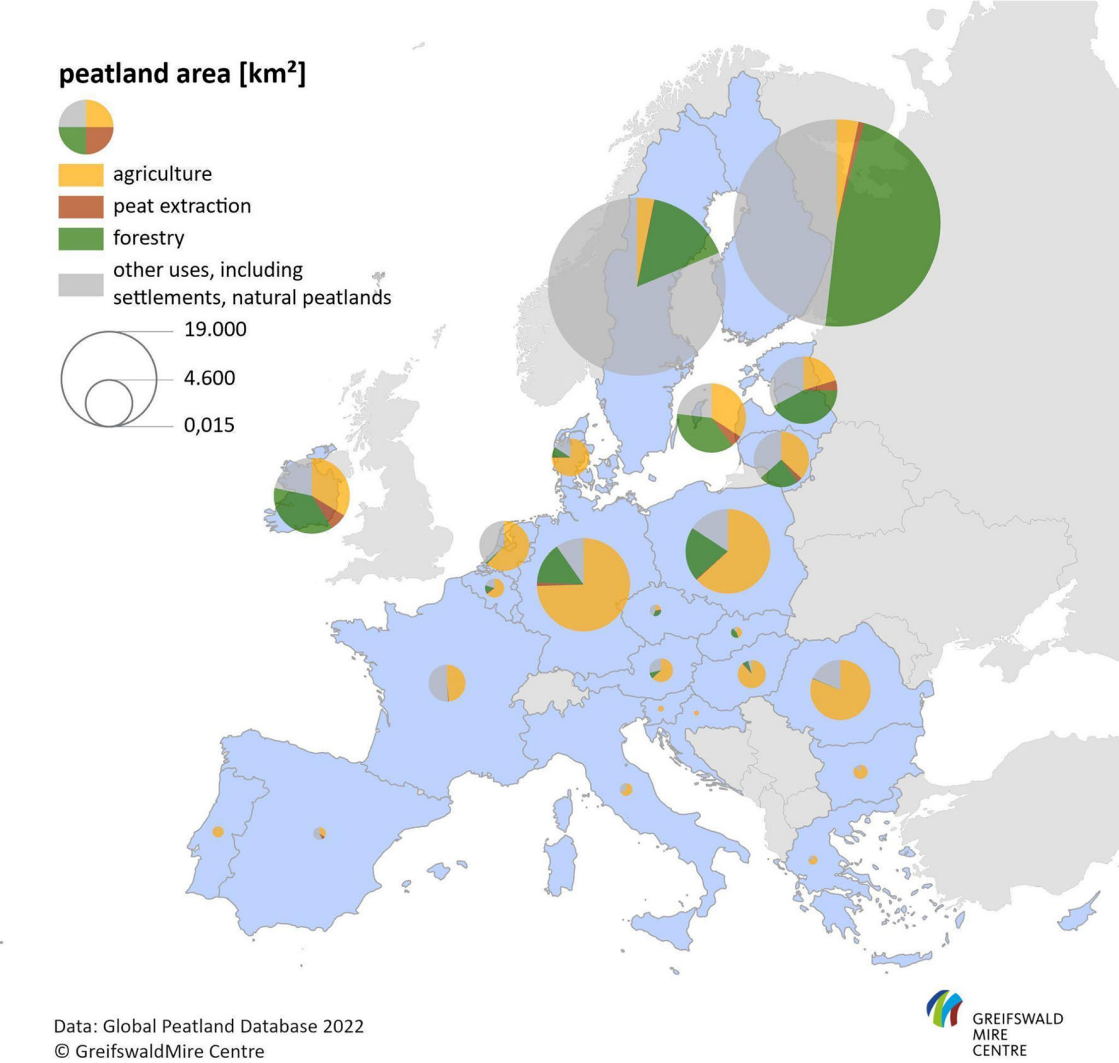
Areal share of peatlands under agriculture, peat extraction, forestry, and other uses in countries of the European Union. Colours refer to different land use types, and the size of the circles reflects total peatland area

In Europe, peatland forestry is mainly found in the north (*i.e.* Finland, Sweden, and Norway), but is also of national importance in the Baltic countries, United Kingdom, Ireland, Poland and Germany (see Fig. 1, data for non-EU countries are not included). Typically, peatlands used for forestry are drained, which leads to a simultaneous increase in CO_2_ and N_2_O emissions through peat decomposition (see above) and CO_2_ sequestration through the growing tree stand; while, the drainage ditches may still emit considerable amounts of CH_4_.

## Climate impact of peatland forestry

Although soil CO_2_ emissions increase after drainage, several studies on drained boreal peatland forests, made with micrometeorological or combined chamber-efflux-litter-production-methods, suggest that carbon sequestration in the tree biomass can exceed the carbon loss from the decomposition of the peat (Lindroth et al. 2007; Meyer et al. 2013; Ojanen et al. 2013; Uri et al. 2017; Minkkinen et al. 2018; Bjarnadottir et al. 2021). In most cases, however, soil C stocks decrease over time, which is the deciding factor when whole rotation climate impacts are considered. Nutrient-poor sites in the boreal zone may accumulate carbon in the soils (Ojanen et al. 2013), but in the absence of a high water table, the fate of this carbon is unclear over a production cycle.

### Measuring greenhouse gas fluxes in peatland forests is challenging

The most common way to measure greenhouse gas fluxes in treeless peatlands has been to place airtight chambers on the surface and to measure the change in the concentration of gases inside the chamber. This commonly applied technique cannot be used in a forest stand as mature trees typically cannot fit inside the chambers. Therefore, the soil carbon balance is estimated by subtracting measured litter production from heterotrophic soil respiration (e.g. Ojanen et al. 2012, 2013; Jovani-Sancho et al. 2021; Uri et al. 2017) where the latter has been measured from trenched plots in which plants and tree roots have been excluded. Simply placing chambers on the forest soil will not provide reliable measurements of fluxes from soil degradation, because the roots of the trees also emit CO_2_ (i.e. autotrophic soil respiration). Intricate chamber set ups are required to distinguish between emissions from the soil (i.e. heterotrophic soil respiration) and from the living tree roots (i.e. autotrophic respiration) (Mäkiranta et al. 2008; Hermans et al. 2022).

The eddy covariance technique (EC), which uses fast measuring gas analysers, requires large flat and homogeneous areas, which are also difficult to find because forestry areas on peatlands are often organised in fairly small blocks. EC is the standard method for ecosystem-level measurements and has a typical uncertainty between 5 and 15% (Burba 2022). The method also allows for the estimation of the soil carbon sink/source by subtracting the modelled biomass increment from the measured net ecosystem exchange. Other soil carbon balance estimation methods include the estimation of soil subsidence through pollen or carbon isotope profiles or by consecutive thickness measurements (Minkkinen et al. 1999; Hooijer et al. 2012; Simola et al. 2012; Sloan et al. 2019). These integrate longer time periods. Obtaining accurate estimates for these carbon balance estimation methods is, thus, challenging.

The above-mentioned studies consider forestry on drained peatlands, but these are based on natural tree stands in the boreal zone. In the temperate zone, sites have more often been drained for agriculture or peat extraction and only later been afforested. Most of the studies do not describe the situation after afforestation of agricultural fields or cutover peatlands, yet this difference in soil management history may lead to different outcomes (Jauhiainen et al. 2023). In addition, the above-mentioned gas exchange studies only give a temporary GHG balance for the study period. *They do not consider the whole forestry cycle, which includes harvesting of the wood (i.e. the removal of sequestered biomass C), decomposition of wood products, and the time needed for stand regeneration.* Harvested sites, especially clear-cut sites, are large carbon sources (Korkiakoski et al. 2019, 2023) until a new stand has regenerated. In addition, only a small proportion of wood products is long-lived as most of the carbon in wood products is lost to the atmosphere in a few years after cuttings (Soimakallio et al. 2016).

### State of knowledge on greenhouse gas exchange in peatland forests

A recent study from Iceland (Bjarnadottir et al. 2021) showed no warming effects of peatland forestry compared to rewetted or wet peatlands. The study area, a poorly drained site, was afforested with a very productive species (Black cottonwood, *Populus trichocarpa*). According to the authors, only further long-term studies and life-cycle assessments will show whether forestry on drained peatlands can really be more climate friendly than wet and healthy peatlands. A recent meta-analysis examining the climate effects of forestry on *shallow* organic soils (< 40–50 cm peat depth) in Scotland showed no warming climate effect (Vanguelova et al. 2018). However, meta-analyses that explore the effects of forestry on *deep* peat soils in the temperate climate region show considerable net emissions of CO_2_ to the atmosphere, at least when the whole life-cycle is considered (Hommeltenberg et al. 2014; Jovani-Sancho et al. 2021). The latter finding confirms the emission factors derived in the 2013 IPCC Wetland Supplement (IPCC 2014). The considerably lower figures for carbon loss from the soil reported in Hermans et al. (2022) show that there can be strong variation across sites, which would suggest that more research on the matter is needed. With respect to the harvesting stage of peatland forestry, selective cutting instead of clear-cutting can lower GHG emissions in a forestry-drained peatland in the boreal climate zone (Korkiakoski et al. 2023). Although the site with partial harvest studied by Korkiakoski et al. (2023) transformed into a CO_2_ sink five years after harvest, peat decomposition continued, releasing almost the same amount of carbon into the atmosphere as was fixed by the trees. Most studies in drained peatland forests show similar results: the ecosystem may be a C sink; whereas, the soil is a C source. Therefore, production forestry, where biomass C is harvested and rapidly lost back to the atmosphere (Soimakallio et al. 2016), will likely result in net C losses in the long-term.

A recent synthesis of site-specific greenhouse gas emissions from drained organic forest soils suggests new emission factors for boreal and cool temperate regions (Jauhiainen et al. 2023). Only one of the derived emission factors, the one for the site type “afforested after peat extraction”, suggests a minor CO_2_ sink (− 86.12 ± 247.34 (SD) g m^−2^ a^−1^). It is based on only one study (featuring 6 sites) and the emission factor for N_2_O for the same site type is actually quite similar but with opposite sign (95.55 ± 32.76 (SD) g m^−2^ a^−1^) when transformed to CO_2_ equivalents (according to IPCC AR6) suggesting overall climate neutrality during the growth phase of the forest. All other emission factors for the discussed site types suggest peatland forests to be overall sources of CO_2_ equivalent emissions to the atmosphere although some categories (e.g. low productivity nutrient-poor sites in the boreal or typical productivity sites in the temperate region (independent of nutrient status)) come out with lower emission factors compared to the IPCC 2014 Tier 1 approach.

Thus, none of the studies discussed above provides a basis to include active afforestation on drained peatlands, especially if managed for production forestry, as a viable option under the NRL.

### Discussion of findings of recent studies lacking scientific rigour

Some recent studies from Latvia do seem to support the idea that afforestation of drained peatlands could be better for the climate than rewetting. However, these studies are inconclusive, have major flaws and biases, and cannot be verified and validated because the methods used are error-prone and descriptions often lack clarity. For instance, Samariks et al. (2023) claim to show that afforestation of peat extraction sites can result in net carbon removals. However, they did not measure all elements of the carbon cycle and failed to clearly distinguish between soil emissions and emissions from tree roots. Instead of analysing, they simply *assumed* that the soil fluxes made up less than half of the measured total flux, independent of changes in management or site conditions, which is unrealistic because the share of soil flux to the total ecosystem flux varies widely (Ojanen et al. 2010). Moreover, the appropriate comparison would have been with a rewetted peat extraction site. Further, after peat extraction, any rehabilitation measures including plant establishment will likely result in net carbon removals because in the first years the biomass stock will build up. Bārdule et al. (2022) suggested that wet peatlands do not achieve lower emissions than drained peatlands. However, they measured only once per month over a period of only four months, during only one vegetation period. Their experimental set up allowed only the emitted CO_2_ flux from the system (soil and litter degradation plus plant respiration) to be measured, but not the sequestration of CO_2_ from the atmosphere during photosynthesis. Moreover, they neglected to account for the fluvial carbon export from the drained sites.

Butlers et al. (2021) reported larger N_2_O and CH_4_ emissions from ‘naturally wet’ sites than from ‘drained’ forests. These findings are not surprising given that the ‘drained’ site in the study had water tables deeper than 60 cm below the soil surface in summer. Again, a full GHG balance can only be assessed when CO_2_ exchange is included as well. Butlers et al. (2022a) claimed that CO_2_ emissions from ‘naturally wet’, nutrient-rich organic forest soils can be larger than those from drained sites but they did not include photosynthesis by ground vegetation in their study and did not quantify CO_2_ release from the tree roots, thus, failing to describe net-CO_2_ exchange appropriately. Without inclusion of the contribution of trees and without a full life cycle assessment, no sensible conclusions about the climate effect of wet vs. drained forested peatlands can be drawn.

Butlers et al. (2022a) attempted to obtain a better understanding of the whole GHG balance by looking at different stages of the harvest cycle and even including the input of litter. Again, the same error-prone methods were used. No distinction is made between root- and soil-derived emissions in the measurements. Instead a regression equation is used, which indicates that slightly more than half of the measured flux is related to decomposition of litter and soil. The equation employed presents a broad relationship that may be helpful to constrain large scale estimates, but was not made to infer site-specific flux values, as was stressed by the original authors of the equation (Bond-Lamberty and Wang 2004). Again, the conclusions are based on limited measurements. They fall far short of the full life-cycle assessment that is essential if reliable data for the effects of forest growth on drained peatlands are to be compared with the situation in rewetted or pristine peatlands. In addition, there were no measurements conducted in intact or rewetted peatlands to provide an appropriate baseline for comparison. Thus, the study results are unsuitable to support far reaching generalisations with regard to afforestation of peatlands as viable options to achieve climate goals under the NRL.

### The need for full production cycle analyses

Most importantly, however, the felling of trees and the subsequent fate of the carbon sequestered in the wood needs to be considered (Ciais et al. 2008). As little as 50% of the actual tree biomass may be extracted during harvest; the remainder is left to decompose on site and within a few years returns as CO_2_ to the atmosphere (Korkiakoski et al. 2019; Leturcq 2020). After a tree is felled, much of its carbon can be stored in wood products. The processing of raw wood for product is estimated to produce (1) waste wood residues and (2) short-lived products (bioenergy, pellets, and pulp and paper (Jasinevičius 2018) of ∼40–60% being subject to fast carbon release (Sokka et al. 2015). Yet a simple carbon balance does not tell the whole story; unlike the carbon stock in soil and peat, not everything made out of wood is climate neutral (Leturcq 2020). As a rule, for a long-lasting product made out of 80-year old wood to be climate cooling, the wood should not be discarded and burnt for at least 40 years after harvest. A product made from 40-year old wood would need to remain for at least 20 years after harvest (Guest et al. 2013; Galimshina et al. 2022). Long-lived harvested wood products are actually quite rare, and harvesting does reduce the total amount of carbon stored in the forest (Soimakallio et al. 2022). In assessing the GHG balance of peatland forestry, complete harvest cycles should be taken into account. Such data are simply absent at the moment.

Since trees grow slowly, no measurements of full growth cycles are yet available and results that span longer time periods are derived from the investigation of chronosequences. Certainly, more chronosequence work is needed, but measurements must be made over multiple years so that variations in weather and other environmental conditions can be integrated into the models. As Vanguelova et al. (2018) have expressed: “There is a clear need for long-term studies using different planting ages (chronosequence studies) to ensure robust results when evaluating the impacts of afforestation and restocking on soil carbon stocks, as short-term impact studies are likely to provide misleading conclusions.”

Overall, Finland's land use sector, for instance, seems to have gradually transitioned from being a CO_2_ sink to a source (Statistics Finland 2022b) driven by increased demand for wood products, slower increase in growing stock, and rising emissions from soil organic matter and litter in drained peat-based forest soils. Siljander et al. (2022) have suggested that the fastest way to strengthen carbon sinks in Finland is to reduce logging, and the Finnish Nature Panel has recommended, among other things, the rewetting and restoration of wetlands and peatlands (Lång et al. 2022).

### Additional possible effects of drained peatland forestry

Peatland forestry on drained sites is more prone to wildfires (Kohlenberg et al. 2018), which will become more frequent and severe in times of climate change with more frequent and more intense droughts in the boreal zone (Walker et al. 2019). Boreal forests in North America have turned from a net sink to a net source of GHG in recent years (Zhao et al. 2021), primarily due to more frequent and more severe fires (Zheng et al. 2023). In addition to the carbon loss from burnt wood, as well as substantial carbon losses from burnt and burning peat layers, should be considered, including the waterborne carbon losses (Liu et al. 2023). In countries with a relatively high density of forest roads, such as Finland, the severity of forest fires on peatland could be increased. This is because large drained peatland areas with forest often have a much lower density of roads compared to areas with mineral ground, thereby making it more challenging to control fires in these peatland regions.

In addition to GHG exchange, the change in albedo and the release of aerosols, together with the lateral exchange of carbon and nitrogen, all add to the total climate effect of ecosystems (Billett et al. 2010). Increased tree cover decreases the albedo effect compared to a treeless mire (Lohila et al. 2010), which leads to local warming (Gao et al. 2014). However, forests are also large sources of biogenic volatile organic compounds (BVOC) and thus may have a considerable cooling impact on the climate (Tunved et al. 2006). In the boreal zone, this impact is similar in magnitude, but opposite to that of the albedo (Kalliokoski et al. 2020).

Aside from site-related atmospheric impacts, forestry on peatland may also have negative impacts on water storage capacity, water quality and nutrients runoff, including loss of organic matter via fluvial pathways, which is subsequently mineralised and the carbon partially returned to the atmosphere (Evans et al. 2016). Rewetting of drained peatlands can also lead to considerable amounts of nitrogen and phosphorus leaching (*e.g.*, Koskinen et al. 2017) but the nutrient-sink function can be rapidly recovered provided further restoration management is implemented (Zak and McInnes 2022). The loss in water storage capacity due to the loss of pore space caused by drainage can manifest itself in greater variations in stream flow and water quality for downstream aquatic ecosystems (Flynn et al. 2021). Intense rainfall events are predicted to increase under climate warming, which can cause mid-term flooding leading to the die-back of trees caused by increased hydrological instability. Indeed, more research to evaluate the trade-off values of wood production, carbon sequestration and emissions, and water storage of natural, drained, and rewetted peatlands like it has been recently published by Makrickas et al. (2023) is urgently required (Manton et al. 2021).

## Conclusions

The most recent IPCC report (IPCC AR6 SYR 2023) outlines in clear language that we must come to grips with proper natural climate solutions since they are paramount to avoid the gravest consequences of climate change and global warming. The Nature Restoration Law (NRL) proposed by the European Commission is a key component to unlock the climate mitigation potential of degraded ecosystems. ***The many open questions and lack of evidence for overall climate benefits of active afforestation on peatlands prohibit its inclusion as a viable climate change mitigation measure in the NRL***. Moreover, the NRL should foster true natural ecosystems wherever possible, particularly where those are demonstrably carbon capture systems. A forest that can only grow when the peat below the trees is drained does not comply with this requirement. ***Afforestation of drained peatlands is not restoration. We cannot restore peatland ecosystems, their flora, their fauna, their functions, by afforestation. The only solution to restore drained peatlands is rewetting.*** The re-introduction of natural mire vegetation can potentially speed-up the restoration process. If trees belong to such an ecosystem, they will regenerate naturally.

## Footnotes

1 *Rewetting* and *restoring/restoration* are often used interchangeably but they are not. *Rewetting* describes the deliberate action of raising the water table on drained soils to re-establish water-saturated conditions. In contrast, *restoration* refers to the full re-establishment of all ecosystem functions that cannot be done directly but only be aimed at facilitating measures, such as rewetting, removal of degraded topsoil, and re-introduction of mire-typical vegetation, etc. See the IPCC Wetlands Supplement (IPCC 2014) for a profound discussion of this terminology.

2 Definitions based on resolution VIII.17 “Guidelines for Global Action on Peatlands” https://www.ramsar.org/sites/default/files/documents/pdf/res/key_res_viii_17_e.pdf, IMCG & IPS Wise Use Guidelines (Joosten & Clarke 2002), and “Mires and Peatlands of Europe” (Joosten et al. 2017).
